# Acute stress-induced alterations in short-chain fatty acids: Implications for the intestinal and blood brain barriers

**DOI:** 10.1016/j.bbih.2025.100992

**Published:** 2025-04-17

**Authors:** Cristina Rosell-Cardona, Sarah-Jane Leigh, Emily Knox, Emanuela Tirelli, Joshua M. Lyte, Michael S. Goodson, Nancy Kelley-Loughnane, Maria R. Aburto, John F. Cryan, Gerard Clarke

**Affiliations:** a APC Microbiome Ireland, University College Cork, Cork, Ireland; b Department of Anatomy and Neuroscience, University College Cork, Cork, Ireland; c Department of Psychiatry and Neurobehavioural Science, University College Cork, Cork, Ireland; d 711th Human Performance Wing, Air Force Research Laboratory, Wright-Patterson Air Force Base, Dayton, OH, USA

**Keywords:** Acute stress, Short-chain fatty acids, Microbiota-gut-brain axis, Barriers, Microbial metabolites

## Abstract

Unravelling the features of the whole-body response to acute stress exposures is critical to understand this neglected building block of chronic stress. A single acute stress exposure rapidly modulates gut-brain axis signalling including intestinal permeability, but the mechanisms are unclear. Microbially-produced metabolites such as short-chain fatty acids (SCFA) are key effectors within the gut-brain axis which can affect gut and brain function. The aim of this work is to determine if acute stress regulates SCFA production in the gut and to understand the associated implications for gastrointestinal and brain barrier function. Stress reduced caecal SCFA concentrations, primarily butyrate and acetate. These SCFAs prevented LPS-induced disruption of gut and brain barrier function in a dose-dependent manner in in vitro models. This functional protection was associated with altered tight-junction abundance and morphology. These results provide a better understanding of the role SCFAs have on barriers following acute stress.

## Graphical abstract

**Figure:**
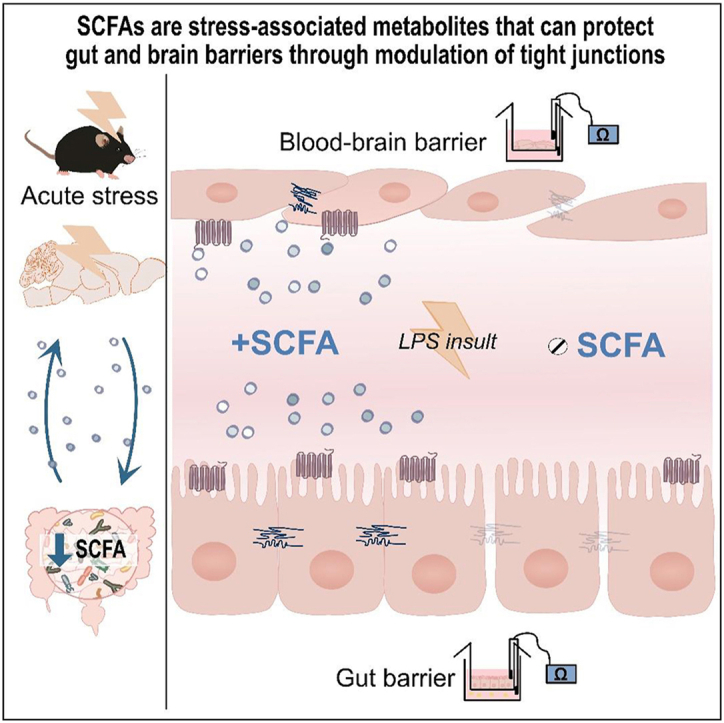

## 1 Introduction

Stress is a complex physiological reaction that can elicit behavioural and physiological changes to restore homeostasis. Two main components of the stress system are the hypothalamic-pituitary-adrenal (HPA) axis and the autonomic nervous system. The stress response depends on the length, intensity, frequency, and modality of the stressor (Herman et al., 2016). Chronic stress leads to significant structural changes in the brain, affecting cognition, memory, the blood-brain barrier, and in the gastrointestinal tract (McEwen et al., 2016; Welcome & Mastorakis, 2020). Chronic stress is a major risk factor for various neuropsychiatric conditions, including depression and anxiety, as well as gut-brain disorders like irritable bowel syndrome (IBS) (Barbaro et al., 2024). Although it is well known that chronic stress disrupts microbiota-gut-brain axis signalling, there is an incomplete understanding of the impact of acute stress at molecular and functional levels in the gut (Leigh et al., 2023). Unravelling the molecular mechanisms linking acute stress exposures to alteration in permeability is critical for understanding how these often-neglected building blocks of chronic stress contribute to health and disease (Atrooz et al., 2021, Gheorghe et al., 2024, Leigh et al., 2023).

In addition, the gut microbiota has emerged not only as an important regulator of the gut permeability, but also as an important modulator of barrier function across the microbiota-gut-brain axis. The microbiota-gut-brain axis is the network involving neural, immune, endocrine, and metabolic pathways between the microbiota, gut and the brain (Aburto & Cryan, 2024; Cryan et al., 2019). Previous studies focused on diets, prebiotics, or probiotics have all been shown to improve intestinal function (Guglielmetti et al., 2020; Krumbeck et al., 2018). Other studies have also demonstrated that the gut microbiota can modulate stress and stress-related disorders in clinical and pre-clinical trials (Ait-Belgnaoui et al., 2012; Beisner et al., 2021; Burokas et al., 2017; Papalini et al., 2019, E. Schneider et al., 2024). While the relationship between diet, the intestinal barrier, and stress related disorders have been well defined, the precise underlying mechanisms linking acute stress, and the gut microbiota remain unclear.

Microbial metabolites, a range of molecules produced by the gut microbiota, are key effectors of the gut microbiota. Among them, short-chain fatty acids (SCFA), mainly acetate, butyrate, and propionate, are the most studied. SCFAs are mainly produced by the microbial fermentation of dietary fibers and are found in the gut lumen, in the circulation, and in the central nervous system (CNS) (O’Riordan et al., 2022). SCFAs have various effects on many aspects of host physiology both locally and at sites distal to their initial production, including regulation of blood pressure (Pluznick, 2017), microglial maturation (Erny et al., 2015; Erny & Prinz, 2017), astrocyte gene expression (Spichak et al., 2021), synaptic plasticity (Rosell-Cardona et al., 2025), and blood-brain barrier integrity (Knox et al., 2022). In addition, administration of SCFAs to mice alleviated the stress induced increase in corticosterone levels, psychosocial stress-induced alterations in reward-seeking behaviour, and increased responsiveness to an acute stressor (van de Wouw et al., 2018). Moreover, in humans, one-week colonic delivery of SCFA mixture attenuated the cortisol response to psychosocial stress compared to placebo (Dalile et al., 2020).

However, although the focus in this field has largely been on microbial regulation of the host stress response, the system is bi-directional and therefore a greater emphasis is needed in understanding how psychological stressors impact the gut and bacterial homeostasis to in turn regulate host physiology (Chang et al., 2024; K. M. Schneider et al., 2023). Acute stress can impact the microbiota community profile and increase gut barrier permeability (Galley & Bailey, 2014; Gheorghe et al., 2024). Stress is also associated with alterations in microbial metabolites (Berding et al., 2023; Gheorghe et al., 2024; van de Wouw et al., 2018), which are more important for the crosstalk between gut and brain particularly at the level of barrier function (Hoyles et al., 2018; Huang et al., 2021; Knox et al., 2022; Li et al., 2016).

Therefore, we aimed to characterise how acute stress alters SCFA and associated metabolite availability in the caecal content of male mice, and to understand how these stress-associated disruptions in SCFAs interface with host physiology to modify gut and brain barrier function using *in vitro* models. This will allow us to better understand the microbial determinants of pro-adaptive versus maladaptive trajectories of the stress response.

## 2 Methodology

### 2.1 Ethics

All animal work carried was approved by the Animal Experimentation Ethics Committee of University College Cork and Health Products Regulatory Authority (HPRA) before beginning this study (Project Authorization AE19130/P160). All experimentation was carried out in accordance with European Directive 2010/63/EU and was also approved by the 711th Air Force Base Institute of Research Intuitional Animal Care and Use Committee (IACUC) the United States Air Force Surgeon General's Office of Research Oversight and Compliance and the Air Force Medical Readiness Agency. Animals were handled and studies were conducted under a program of animal care accredited by the Association for Assessment and Accreditation of Laboratory Animal Care (AAALAC) International.All experiments were performed in compliance with the Animal Welfare Act in accordance with the National Research Council's 2011 Guide for the Care and Use of Laboratory Animals (in compliance with Department of Defense Instruction 3216.1). The experiments in this report were conducted in a facility accredited by bAAALAC.

### 2.2 Studies in animals

Animal procedures were as previously described (Gheorghe et al., 2024). For germ-free mice, male C57/BL6 mice breeding pairs were acquired from Taconic Biosciences. Germ-free, colonized-germ-free, and conventional mice were housed 2–4 mice/cage under a 12-h light/dark cycle and maintained on ad libitum autoclaved water and autoclaved, pelleted diet (Special Diet Services). Housing conditions for germ-free, conventional, and colonized germ-free adhered to the same environmental conditions of temperature (21 ± 1 °C) and humidity (55 %–60 %). Germ-free mice were housed in gnotobiotic flexible-film isolators. Colonized germ-free mice were born and maintained as germ-free mice in gnotobiotic flexible-film isolators until postnatal day 21 when they were removed from the isolators and, for the remaining duration of this study, re-located to the standard animal facility and housed in wire-top cages that contained used-bedding from age- and sex-matched conventional mice, which has been shown previously to confer a gut microbiota composition equivalent to conventional mice. For all other studies, adult male C57/BL6 (20–29 g) mice purchased from Envigo were group-housed 2–3 per cage and maintained on a 12/12 h dark–light cycle with a room temperature of 22 ± 1 °C on ad libitum water and autoclaved, pelleted diet (Envigo).

### 2.3 Acute restraint stress

The acute restraint stress procedure was performed using a clean perforated polypropylene screw-cap 50 mL conical tube as previously described (Lyte et al., 2020). Cages were randomly assigned to either non-stress or stress groups. Each mouse that underwent stress was placed into the 50 mL tube and restrained for 15 min. After 15 min of restraint stress, mice were removed from the restrainer and either transported immediately to the cull room or returned to their home cage and left undisturbed for 45 min. To control for the variable of transport stress, mice from the non-stress control group were placed into new cages containing fresh bedding and transported the same distance before entering the cull room. Upon entering the cull room, mice were rapidly decapitated and caecal contents were collected.

### 2.4 Caecal SCFA quantification

SCFA quantification was conducted by Metabolon Inc. using ultra-high performance liquid chromatography (UHPLC)-tandem mass spectrometry (MS), as described below. Samples were prepared using the automated MicroLab STAR system from Hamilton Company. Mouse caecal samples were spiked with stable labelled internal standards and were homogenized and subjected to protein precipitation with an organic solvent. After centrifugation, an aliquot of the supernatant was derivatized. The reaction mixture was diluted, and an aliquot was injected onto an Agilent 1290/AB Sciex QTrap 5500 LC MS/MS system equipped with a C18 reversed phase UHPLC column. The mass spectrometer was operated in negative mode using electrospray ionization (ESI). The peak area of the individual analyte product ions was measured against the peak area of the product ions of the corresponding internal standards. Quantitation was performed using a weighted linear least squares regression analysis generated from fortified calibration standards prepared immediately prior to each run. LC-MS/MS raw data were collected and processed using AB SCIEX software Analyst 1.6.2. Data reduction was performed using Microsoft Excel for Office 365 v.16.

### 2.5 Untargeted metabolomics

Untargeted metabolomics presented in this paper comprise post-hoc analysis of a dataset previously published (caecal contents, https://doi.org/10.21228/M89M7J; Project ID: PR001945; colonic mucosa, https://doi.org/10.21228/M89M7J; Project ID: PR001945) (Gheorghe et al., 2024). Caecal contents and colonic mucosa metabolomics were conducted by Metabolon Inc. using ultrahigh performance liquid chromatography-tandem mass spectrometry, as described below. Samples were prepared using the automated MicroLab STAR system from Hamilton Company. Several recovery standards were added prior to the first step in the extraction process for QC purposes. To remove protein, dissociate small molecules bound to protein or trapped in the precipitated protein matrix, and to recover chemically diverse metabolites, proteins were precipitated with methanol under vigorous shaking for 2 min (Glen Mills GenoGrinder, 2000) followed by centrifugation. The resulting extract was divided into five fractions: two for analysis by two separate reverse phase (RP)/UPLC-MS/MS methods with positive ion mode electrospray ionization (ESI), one for analysis by RP/UPLC-MS/MS with negative ion mode ESI, one for analysis by HILIC/UPLC-MS/MS with negative ion mode ESI, and one sample was reserved for backup. Samples were placed briefly on a TurboVap (Zymark) to remove the organic solvent. The sample extracts were stored overnight under nitrogen before preparation for analysis.

### 2.6 Cell lines

Human carcinoma cells (T84; CCL-248) and murine brain endothelial cells (bEnd.3; CRL-2299) were purchased from ATCC (American Type Culture Collection, Middlesex, UK). Both cell lines were cultured in Dulbecco's Modified Eagle Medium: Nutrient Mixture F-12 (containing 10 % heat-inactivated fetal bovine serum (Sigma-Aldrich, F9665) and 1 % penicillin/streptomycin). Cells were maintained at 37 °C and 5 % CO_2_. The media was changed every 2–3 days. Cells under passage 15 (T84) or passage 20 (bEnd.3) were used for all experimentation. T84 cells were seeded at 1∗10^5^ cells/well, while bEnd.3 cells were seeded at 4.5∗10^3^ cells/well, and grown to confluence before starting treatments. Confluent cells were treated in the apical compartment with microbial metabolites diluted in complete media at a range of concentrations (T84; 0, 1, 10, 100, 1000, 10000 μM bEnd.3; 0, 0.1, 0.5, 1, 10, 100, 1000 μM). After 24hrs, LPS *Escherichia coli* O26:B6 (Sigm-Aldrich, #L8274) diluted in media was added to the cells. Control wells and LPS control wells were treated with 0 μM metabolite. T84 cells have been previously demonstrated to exhibit resilience to supraphysiological concentrations of LPS (Kuo et al., 2015). LPS insult was optimized to be sufficient enough to induce a marked increase in permeability, but did not affect cell viability/dead (Supplementary Fig. 6).

### 2.7 Chemicals

The SCFA metabolites were purchased and dissolved directly in endotoxin-free milliQ water and stored in −80 °C before further dilution into complete media for treatment the same day. The gut microbiota metabolizes dietary fiber into the selected SCFAs through major pathways including Wood-Liungdahi, acylate, succinate, and propanediol pathways (Fig. 2).

### 2.8 Trans-epithelial/endothelial electrical resistance (TEER)

TEER is a technique used to measure the integrity of tight junction dynamics in endothelial and epithelial cell monolayers and was used as an index of paracellular and transcellular permeability. Either T84 or bEnd.3 cells were seeded on 0.4 μm 24 well plate polyethylene terephthalate transwell inserts (surface area 0.33 cm2, pore size 0.4 μm; Grenier Bio-one #662641). Once confluent, TEER was measured with a World Precision Instrument Epithelial volt/Ohm Meter2 machine and chopstick electrodes. TEER (Ωcm2) was calculated by subtracting the value obtained by the cell-free inserts, and then multiplied by the surface area of the insert. TEER was measured at time 0, 24 and 48 h after treatment with metabolites that were added in the apical compartment. Values obtained from cell-free inserts were subtracted from the total values and expressed as percentage of control.

### 2.9 Permeability FITC assay

Following the final TEER assessment, the transwell plates were washed once with 1x HBSS. The transwell inserts were changed to a new plate with 500 μl of 1xHBSS, and 4 kDa Fluorescein isothiocyanate (FITC) dextran 800 μg/ml (Sigma-Aldrich #FD4) in 1xHBSS was added to the apical compartment of transwells to study paracellular permeability assay. After a 60min incubation at 37 °C, the transwell inserts were removed and 100 μl per well were collected from the basolateral compartment in triplicates in a 384-well plate. The plate was then read at 485/535 nm. Using a standard curve, the μg/mL of FITC was calculated and represented as percent of the untreated control.

### 2.10 Immunofluorescence imaging

T84 cells were seeded at 1∗10^5^ cells/well in μ-slide 8 well plates from IBIDI (#80826) and grown to confluency in complete media. Butyrate, propionate, and acetate at 10 mM were added to the media before the addition of LPS. Upon completion of the experiment, cells were fixed with 10 % ice cold 3.7 % PFA or TCA for 15min then washed 3x with PBS. Cells were then permeabilized with 0.3 % Triton X-100 in PBS for 10min at room temperature and blocked with 10 % normal donkey serum in PBS for 30min at room temperature. Primary antibodies (ZO-1 #61–7300, Alexa Fluor™ 647 Phalloidin #A22287 ThermoFisher Scientific) were diluted 1:150 in 2 % normal donkey serum and incubated overnight at 4C. Cells were then washed in PBS 3x before adding the secondaries diluted in PBS at 4 °C overnight. Cells were then washed and kept in PBS for confocal imaging.

### 2.11 Quantitative PCR

Cells were seeded in 24 well plates and grown for 10 days. At completion of the experiment, RNA was isolated from the tissue using the GenElute Mammalian Total RNA Miniprep kit from sigma (#RTN70-1 KT). RNA was reverse transcribed to cDNA using a high-capacity cDNA reverse transcription kit from Thermo Fisher. Gene expression was assessed by fluorescent real time quantitative PCR using the LightCycler 480 Real-Time PCR System. Taqman primer sequences for *Ffar2, Ffar3, tight junction protein 1* for detection of mRNA were used. Each sample was run in duplicate and cycle threshold (CT) values were used for gene expression calculations, and all CT values were normalized to the expression of *Gadph* and *hprt1.* Relative gene expression was calculated using equation 2ˆ-ΔΔCT and expressed as fold change normalized to the untreated controls (Livak & Schmittgen, 2001).

### 2.12 MTT cell viability assay

T84 cells were seeded at 1∗10^5^ cells/well while bEnd3 cells were seeded at 4.5∗10^3^ cells/well. The MTT plates were treated the same way as the transwell plates. At the completion of the experiment, the media was removed from the cells and 50 μL of 0.5 mg/ml MTT (Sigma Merk #M5655-500 MG) was added to each well. The plate was covered and placed in the 37 °C incubator for 2–3hrs to allow crystals to form. The MTT was then removed from the wells and 100 μL of dimethyl sulfoxide was added to each well. The plate was placed on a plate shaker for 15–20min to allow the crystals to dissolve. The plate was then read at absorbance 570 nm. Percent viability compared to the untreated wells was calculated by subtracting the average reading of the blank wells and then dividing this number by the average reading of the untreated wells minus the blank and then multiplied by 100 for percentage.

### 2.13 Live/dead assay

Live/Dead assay was determine using a LIVE/DEAD™ Viability/Cytotoxicity Kit, for mammalian cells (#L3224, ThermoFisher Scientific). DMSO (Dimethyl sulfoxide, #D8418 Sigma Aldrich) was added as a positive control. Fluorescence was read using a Biotek Synergy H1 plate reader 700 equipped with Gen5 software (Biotek).

### 2.14 Quantification and statistical analysis

Statistical analyses for *in vivo* experiments were performed in R (version 4.2.0) using the Rstudio GUI (version 2022.2.2.485). Studies were analyzed using general linear models with the rstatix package or using functions available through https://github.com/thomazbastiaanssen/Tjazi (Bastiaanssen Quinn, and Loughman 2023). Where appropriate post-hoc pairwise comparisons were performed using Tukey's procedure.

The *in vitro* experimental data was expressed as mean ± SEM, n = biological replicates. At least two independent experiments were performed with at least three biological replicates in each experiment. Grubbs test was used to remove any outliers from data sets. Data was analyzed by two-way analysis of variance ANOVA for treatment (Untreated, and metabolites concentrations or type of metabolites) and LPS insult followed by Tukey-adjusted post hoc testing; or Dunnett's multiple comparison to the untreated groups using SPSS Statistics software (IBM, Armonk, NY, US). A p-value of less than 0.05 was considered significant.

## 3 Results

### 3.1 Caecal butyric, acetic and propionic acid are reduced by acute restraint stress

**Fig. 1:**
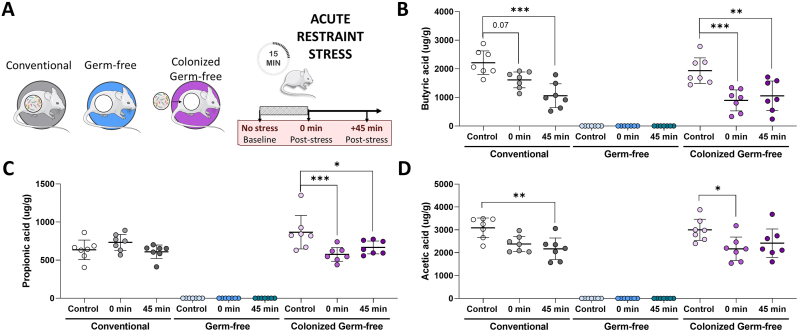
*Short-chain fatty acid content in the gut lumen is reduced by acute restraint stress.* (A) Experimental design for *in vivo* experiment: conventional, germ-free, and colonized germ-free mice underwent 15-min restraint. Mice were euthanized either naive (control), immediately post-stress, or recovered for 45 min post-stress. Caecal content concentrations of (B) butyric acid, (C) acetic acid and (D) propionic acid. Data as mean ± SEM, overlaid individual values (*n* = 7 mice/group); analyzed by two-way general linear modeling followed by Tukey-adjusted post hoc comparisons. ∗p < 0.05, ∗∗p < 0.01, and ∗∗∗p < 0.001.

We first assessed SCFA concentrations in the caecal contents of male conventional, germ-free (GF) and colonized GF mice before, immediately after and 45 min after acute restraint stress (Fig. 1A). While all SCFAs were differently expressed based on microbial status, only butyric (Fig. 1B; F_4,54_ = 6.84, p < 0.001), propionic (Fig. 1C; F_4,54_ = 5.87, p < 0.001), and acetic (Fig. 1D; F_4,54_ = 3.06, p = 0.024) acid concentrations exhibited significant interaction effects between stress exposure and microbial status. In conventional mice, both butyric and acetic acid were reduced 45 min following acute restraint, with no effect on propionic acid. In colonized GF mice, butyric and propionic acid were significantly reduced both immediately and 45 min following stress, while acetic acid was reduced only immediately following acute restraint. These data indicate that stress is associated with reduced content of some SCFAs in the gut lumen. While the response to stress is similar for butyrate and acetate between conventional and colonized GF mice, our results indicate that the response dynamics for propionate are disrupted in mice born GF and subsequently colonized. All other SCFAs assessed are presented in Supplementary Fig. 1 and were not significantly altered by stress. As expected, none of the SCFAs assessed were detectable in GF mice.

**Fig. 2:**
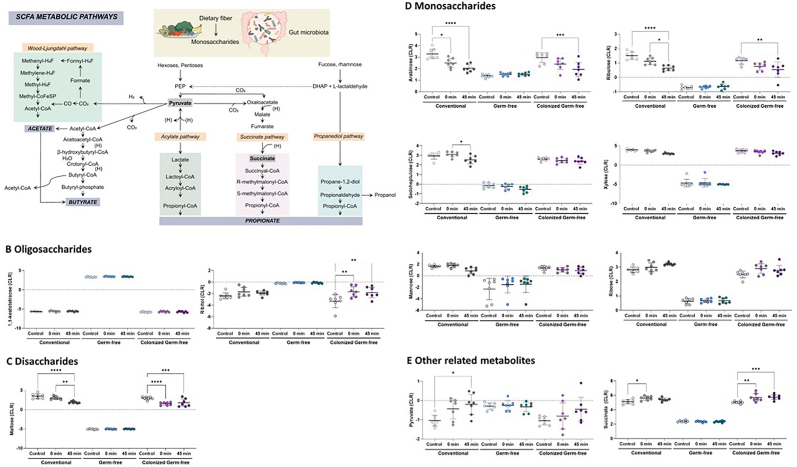
Short-chain fatty acid substrates in the gut lumen are reduced by acute restraint stress. (A) Dietary fiber is metabolized by the gut bacteria into various metabolites such as butyrate, acetate, and propionate. Major pathways include Wood-Liungdahi, acylate, succinate, and propanediol pathways. Metabolites tested in this study are highlighted in purple. Caecal content concentrations of (B) oligosaccharides, (C) disaccharides, (D) monosaccharides and (E) other related metabolites. Data as mean ± SEM, overlaid individual values (*n* = 7 mice/group); analyzed by two-way general linear modeling followed by Tukey-adjusted post hoc comparisons. ∗p < 0.05, ∗∗p < 0.01, and ∗∗∗p < 0.001. (For interpretation of the references to colour in this figure legend, the reader is referred to the Web version of this article.)

### 3.2 Caecal disaccharide and monosaccharide content are reduced by acute restraint stress

Since the majority of SCFA content in the gut lumen is derived from dietary fibre degradation by bacteria (Fig. 2A), we next examined whether acute restraint stress altered these dietary substrates. Some dietary oligosaccharides were altered by acute restraint stress such as 1,1-kestotetraose and ribitol (Fig. 2B; F_2,54_ = 4.16, FDR = 0.065 and F_2,54_ = 8.09, FDR = 0.007 respectively). Post-hoc assessment indicated that ribitol was increased following stress in colonized GF caecal contents. Other oligosaccharides detected were not significantly altered by stress (Supplementary Fig. 2A). Disaccharide maltose exhibited a significant interaction between stress and microbial status (Fig. 2C; F_4,54_ = 9.12, FDR = 0.003) where stress significantly reduced its expression in colonized mice only, while other disaccharides detected did not exhibit any effects of stress (Supplementary Fig. 2B). In contrast, all but one monosaccharide (mannose) were significantly altered by stress (Fig. 2D): arabinose (F_4,54_ = 6.02, FDR = 0.018) and ribulose (F_4,54_ = 5.57, FDR = 0.024) exhibited significant interactions between stress and microbial status while sedoheptulose (F_2,54_ = 7.24, FDR = 0.011) and xylose (F_2,54_ = 6.33, FDR = 0.017) were significantly reduced by stress overall. Ribose was uniquely increased following stress (F_2,54_ = 5.46, FDR = 0.029). These data suggest an almost universal reduction in monosaccharides following acute restraint stress.

Additionally, caecal concentrations of some metabolites involved in energy metabolism (Fig. 2E) including pyruvate (F_2,54_ = 5.40, FDR = 0.029) and succinate (F_4,54_ = 4.23, FDR = 0.056) were increased in colonized mice, while others remained unchanged (Supplementary Fig. 2C). Interestingly, other metabolites produced by microbes from monosaccharides were reduced by acute restraint stress (Supplementary Fig. 2D) including arabonate (F_2,54_ = 13.98, FDR>0.001) and ribonate (F_2,54_ = 5.46, FDR = 0.029), indicating reduced concentrations of microbial products from monosaccharides is not restricted to short-chain fatty acids and that there may be more global shifts in microbial metabolism in response to stress.

### 3.3 Colonic mucosal metabolites involved in energy metabolism are reduced by acute restraint stress

Since short-chain fatty acids are a key energy substrate for intestinal epithelial cells (Supplementary Fig. 3A) (den Besten et al., 2013), we examined whether metabolites involved in energy metabolism were altered by acute restraint stress in the colonic mucosa. While we did not observe differences in the levels of TCA cycle substrates glucose and pyruvate (Supplementary Fig. 3B), microbially-associated TCA cycle substrate (Supplementary Fig. 3C) 2-methylcitrate was reduced immediately following stress in conventional mice (F_2,54_ = 10.66, FDR = 0.008) and two TCA cycle components (Supplementary Fig. 3D), succinylcarnitine and oxalate, were also reduced following stress. Succinylcarnitine, altered in conditions associated with physiological stress, was significantly reduced in conventional mice only (F_2,54_ = 9.59, FDR = 0.012), whereas oxalate, known to induce oxidative stress and increased by physiological stress, was reduced by stress in germ-free mice (F_2,54_ = 8.14, FDR = 0.024). Overall, very few energy-related metabolites were significantly altered by acute stress in the colonic mucosa.

### 3.4 Specific concentrations of short-chain fatty acids protect gut barrier integrity

**Fig. 3:**
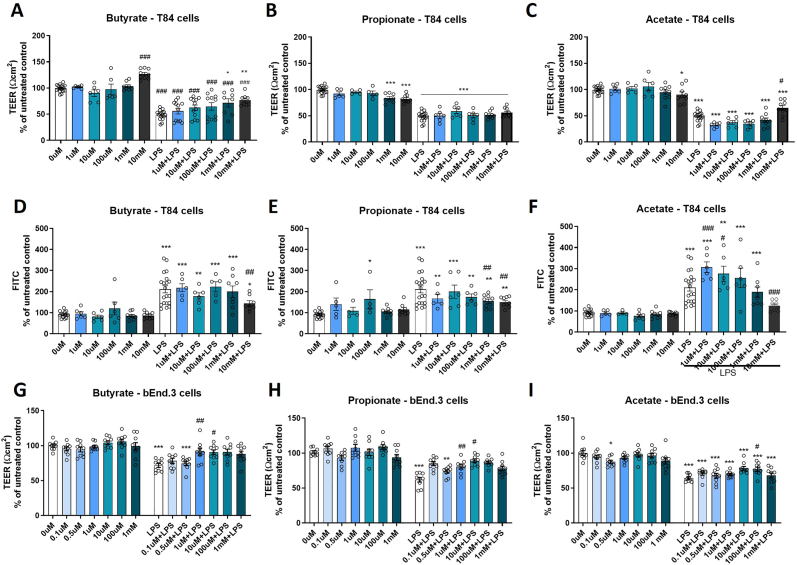
Short chain fatty acids protect LPS-induced barrier disruption. (A–C) Transepithelial electrical resistance of T84, and (G–I) transendothelial electrical resistance bEnd.3 cells monolayers, and (D–F) FITC dextran 4 kDa permeability assay in T84 cells following 48hr (A,D,G) butyrate, (B,E,H) propionate, and (C,F,I) acetate treatment with and without LPS for the last 24hrs of incubation (1 μg/mL for bend3 cells; 500 μg/mL for T84 cells). All metabolites in panels A–C, and D-F were tested on the same plates with the same controls. Data are mean ± SEM two-way ANOVA followed by Tukey's post hoc. ∗p < 0.05, ∗∗p < 0.01, ∗∗∗p < 0.001 metabolite compared to 0 μM control, #p < 0.05, ##p < 0.01, ###p < 0.001 compared to 0 μM LPS.

To assess the effects of SCFAs altered by stress on gut barrier integrity, T84 cells were exposed to butyrate, propionate, or acetate over 48hrs, with and without LPS insult, and over the concentration range reported in the colon, where SCFA can reach a concentration over than 20 mM (https://hmdb.ca/; (Cong et al., 2022). Transepithelial/transendothelial electrical resistance (TEER) measurements indicated the transcellular and paracellular permeability (Srinivasan et al., 2015). In all SCFAs experiments, LPS insult disrupted barrier integrity resulting in a reduction of TEER [LPS, F(1,102) = 553.696, p < 0.001] (Fig. 3A–C for experiments with butyrate, propionate and acetate). There was also an effect of the treatment with butyrate on TEER [Butyrate; F(5,127) = 12.202 p < 0.001; Propionate; F(5,99) = 186.189, p = 0.041; and an interaction between metabolite and LPS with propionate, and acetate experiments in TEER [Propionate; ∗LPS F(5,99) = 449.776, p < 0.001; Acetate∗LPS; F(5,102) = 8.616, p < 0.001; and a trend in Butyrate∗LPS; F(1,116) = 1.909 p = 0.098]. Specifically, butyrate 10 mM increased TEER on T84 cells compared to the 0 μM control wells. Butyrate 1 mM, and 10 mM, and acetate 10 mM increased TEER in LPS-treated cells [Butyrate, 1 mM p = 0.002, 10 mM p < 0.001; Acetate 10 mM p = 0.013]. Propionate, however, did not significantly alter TEER compared to the LPS control.

Additionally, FITC assays were performed to study paracellular permeability. We observed that LPS increased FITC, disrupting gut barrier integrity in all experiments [LPS, F(1,98) = 136.445, p < 0.001] (Fig. 3D–F for experiments with butyrate, propionate and acetate). Pre-exposure to SCFA metabolites prevented LPS-induced disruption [Butyrate, F(5,101) = 2.704, p = 0.025; Acetate, F(5,98) = 4.285, p = 0.002, and there was an interaction between metabolite and LPS with Propionate∗LPS, F(5,100) = 3.152, p = 0.01; and Acetate; ∗LPS F(5,98) = 4.498, p = 0.001. Of these, butyrate and acetate at 10 mM, and propionate at 1 mM and 10 mM reduced FITC on LPS-treated T84 cells compared to LPS-treated group. In contrast, acetate at 1 μM and 10 μM increased paracellular permeability compared to the LPS-treated group. Of interest, butyrate, propionate and acetate at the concentrations used did not alter cell viability of T84 cells (Supplementary Fig. 4). In summary, pretreatment with butyrate (1, 10 mM), propionate (10 mM), and acetate (10 mM) significantly prevented gut barrier permeability induced by LPS insult.

### 3.5 Specific concentrations of short-chain fatty acids protect brain barrier integrity

To understand if the alteration in relative abundance of SCFAs could also impact brain barrier physiology, bEnd.3 cells were exposed to a range of concentrations of butyrate, propionate, or acetate over 48hrs with and without LPS insult. The concentration of acetate in adult blood under normal conditions is reported up to 69.14 μM while butyrate and propionate are reported up to 1.0 (0.3–1.5) μM and 1.6 ± 1.2 μM respectively (https://hmdb.ca/). In butyrate, propionate and acetate experiments, there was an observed LPS effect on barrier integrity as indicated by TEER [Butyrate; F(1,63) = 43.101, p < 0.0001, Propionate; F(1,93) = 157.006, p < 0.0001, Acetate; F(1,94) = 261.460, p < 0.0001] (Fig. 3G and H). There was also an effect of metabolite with butyrate, propionate, and acetate on TEER [Butyrate; F(5,63) = 3.876 p < 0.01, Propionate; F(5,93) = 9.391, p < 0.0001, Acetate; F(5,94) = 3.906, p < 0.01]. Of these, only acetate at 0.5 μM significantly reduced TEER compared to the 0 μM control wells. Of interest, there was an interaction between metabolite and LPS with butyrate, propionate, and acetate experiments [Butyrate; F(5,63) = 1.507 p < 0.05, Propionate; F(5,93) = 4.668, p < 0.001, Acetate; F(5,94) = 2.896, p < 0.05]. Butyrate, propionate and acetate at the concentrations used did not alter cell viability of bEnd.3 cells (Supplementary Fig. 4). Pretreatment with butyrate (1, 10 μM), propionate (0.1, 1, 10, 100 μM), or acetate (100 μM) before addition of LPS significantly increased TEER compared to the LPS-treated control cells.

### 3.6 Short-chain fatty acids increase tight junction protein abundance and induce ruffles

**Fig. 4:**
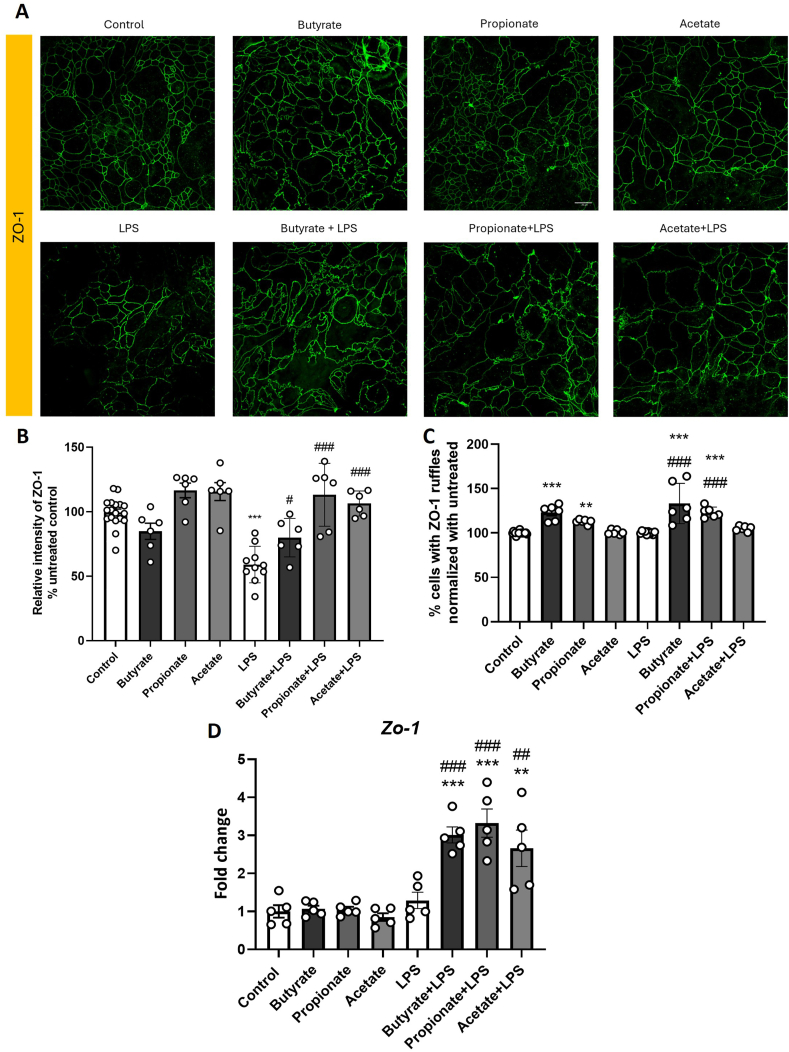
Short-chain fatty acids increase ZO-1 protein abundance and induces ruffles (A) Representative 60x confocal images of ZO-1 staining in T84 cells following 48hr butyrate, propionate, and acetate treatment with and without LPS for the last 24hrs of incubation. (B) Relative intensity of ZO-1, (C) ZO-1 ruffles in T84 cells following 48hr, (D) ZO-1 expression. Data are mean ± SEM two-way ANOVA followed by Dunnett's post hoc. ∗p < 0.05, ∗∗p < 0.01, ∗∗∗p < 0.001 metabolite compared to 0 μM control, #p < 0.05, ##p < 0.01, ###p < 0.001 compared to 0 μM LPS. Scale bar: 20 μm.

To elucidate the mechanism through which SCFAs are able to protect the gut barrier, immunohistochemistry experiments were performed in order to visualize and examine tight junctions on T84 cells (Fig. 4A). Tight junctions are known to play a role in intestinal barrier function (Horowitz et al., 2023). Confocal microscopy revealed impairment of barrier integrity by LPS insult, reducing zonula occludens-1 (ZO-1) protein abundance on T84 cells in all SCFAs experiments [LPS, F(1,56) = 48.322, p < 0.001) (Fig. 4B). Pretreatment with butyrate, propionate, or acetate at a concentration of 10 mM, chosen based on previously shown preventive effects against LPS-induced impairment in T84 cells, significantly protected against the LPS-induced decrease in ZO-1 protein levels (Butyrate, p = 0.049, Propionate, p < 0.001; Acetate, p < 0.001. Additionally, we analyzed non-linear ZO-1 morphologies, called ruffles (Fig. 4C) (Lynn et al., 2020). Butyrate and propionate increased ruffling of the predominant ZO-1 protein with and without LPS (without LPS, Butyrate P < 0.001; Propionate p = 0.005; with LPS, Butyrate p < 0.001; Propionate p < 0.001). Acetate did not increase the number of ruffles. We also analyzed actin directionality in T84 cells, but neither LPS nor SCFA treatment altered this parameter (Supplementary Fig. 5). To confirm these previous results, we analyzed *ZO-1* gene expression by qPCR. Butyrate, propionate, and acetate increased *Zo-1* gene expression in LPS treated cells (Butyrate p < 0.001; Propionate p < 0.001; Acetate p = 0.003; Fig. 4 D).

### 3.7 Short-chain fatty acids modulate the gene expression of their receptors

**Fig. 5:**
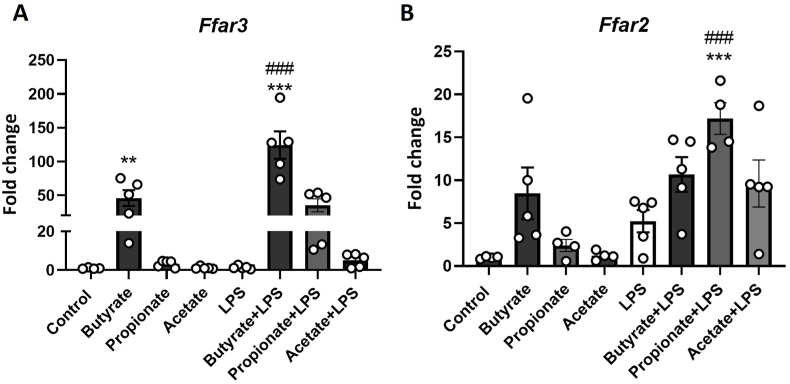
Short-chain fatty acids modulate gene expression of tight junction and their receptors Gene expression *Ffar3* (A) and *Ffar2* (B) in T84 cells following 48hr butyrate, propionate, and acetate treatment with and without LPS for the last 24hrs of incubation. Data are mean ± SEM two-way ANOVA followed by Dunnett's post hoc. ∗p < 0.05, ∗∗p < 0.01, ∗∗∗p < 0.001 metabolite compared to 0 μM control, #p < 0.05, ##p < 0.01, ###p < 0.001 compared to 0 μM LPS.

Since SCFAs have affinity to the receptors FFAR3 and FFAR2, we determined the gene expression of these receptors to understand the mechanism underlying SCFA effects. In *Ffar3* gene expression, there is a LPS, treatment and interaction effect [LPS, F(1,38) = 18.959, p < 0.001; Treatment, F(3,38) = 36.505, p < 0.001; LPS∗Treatment, F(3,38) = 7.561, p < 0.001, Fig. 5A). Butyrate significantly increased *Ffar3* with and without LPS and propionate trend to increase it with LPS insult (without LPS, Butyrate p = 0.016; with LPS, Butyrate p < 0.001; Propionate p = 0.083). In *Ffar2* gene expression, there is a LPS, treatment and interaction effect [LPS, F(1,35) = 27.528, p < 0.001; Treatment, F(3,35) = 5.364, p = 0.005; LPS∗Treatment, F(3,35) = 66.675, p = 0.022, Fig. 5B). Butyrate trend to increase *Ffar2* without LPS (without LPS, Butyrate p = 0.080), and propionate increased it with LPS (with LPS, Propionate P = 0.001).

## 4 Discussion

Understanding the mechanisms underlying acute stress-induced effects on host physiology has been somewhat neglected to date in the context of microbiome-stress interactions. In this study we demonstrate, to what is for our knowledge the first time, that short-term stressor exposure is sufficient to disrupt the metabolic activity of the gut microbiota by reducing SCFA levels in the caecal content, and that these SCFAs can protect gut and blood-brain barriers from an insult. Interestingly, SCFAs regulated transcellular and paracellular permeability of both gut and brain barriers and prevented LPS-disrupting effects in a dose- and compound-dependent manner. Our results provide additional mechanistic insight into previous research showing that exposure to this duration of acute psychosocial stressor can impact intestinal permeability *in vivo* (Gheorghe et al., 2024).

Since increased paracellular intestinal permeability can be due to tight junction dysfunction (Horowitz et al., 2023), we also studied it tight-junction proteins integrity by confocal microscopy. Wang and colleagues showed that butyrate, but not acetate and propionate can regulate synaptopodin, an actin-binding protein (Wang et al., 2020). Although, we have not observed differences in actin directionality, we demonstrated a protective effect of all SCFAs against LPS-induced reduction in ZO-1 protein in colonocytes. ZO-1 is a key tight junction protein essential for maintaining the integrity of the intestinal barrier. A reduction in ZO-1 levels leads to increased paracellular permeability, contributing to barrier dysfunction (Chelakkot et al., 2018).

In addition, our previous research demonstrated that butyrate and propionate influence cytoskeletal proteins by inducing spike formations in the tight junctions of brain endothelial cells (Knox et al., 2022). Here, we demonstrate that this effect is also replicated in the gut, with morphological changes in the tight junctions manifesting as ruffles. The formation of ruffles in tight junction proteins has been observed in various endothelial and epithelial cell types (Lynn et al., 2020). Ruffling has been contrastingly correlated with both increased (Saeedi et al., 2015) and reduced permeability (Tokuda et al., 2014). In our study, the formation of ruffles, along with the increased percentage of tight junction proteins, may be related with the protective effect of specific SCFAs observed in TEER and FITC measurements.

The mechanisms of action underlying the effects of SCFAs could involve G-protein complex receptors (GPCR), mainly GPR41 or FFAR3, and GPR43 or FFAR2. In particular, we found upregulated expression of FFAR genes in T84 cells following SCFA treatment. These receptors play a critical role in maintaining gut integrity, as demonstrated by FFAR2 and FFAR3 knock-out mice, which exhibit increased gut permeability, reduced levels of tight junction proteins, and heightened inflammation, ultimately affecting gut and brain function (Mishra et al., 2020; 2024). Additionally, these receptors may interact with the TLR4-Myd88-NF-kB pathway, inhibiting the signalling cascade, as previously demonstrated in studies involving SCFAs or SCFA-producing bacteria (Guo et al., 2023; Liu et al., 2023, Zheng et al., 2020). To disrupt barrier integrity, we used the pro-inflammatory LPS *Escherichia coli* O26:B6 as a Gram-negative bacterial cell wall component that can be recognized as a pathogen associated molecular pattern (PAMP) by toll-like receptors (TLRs). This LPS can activate TLR4, widely expressed in gut and brain barriers, and trigger Myd88-dependent and independent pathways, resulting in the nuclear translocation of NF-kB or IRF3 that will activate inflammatory response and compromise both transcellular and paracellular gut barrier integrity (Stephens & von der Weid, 2020). These findings suggest that the impact on gut barrier integrity may occur through a receptor-mediated mechanism, with FFAR3 playing a key role in promoting the expression of tight junction proteins (such as ZO-1), enhancing the resilience of the cellular barrier against potential disruptors or acting along the LPS-related cascade to restore barrier function.

We found that acetate can have either protective or disruptive effects on the gut and brain barriers, depending on the dose. Previous studies have shown that a Mediterranean diet, rich in fiber, can improve intestinal barrier integrity through the production of these compounds (Seethaler et al., 2022). For instance, Hoyles et al., described that butyrate and propionate, but not acetate prevented LPS-induced disruption in the BBB *in vitro* (Hoyles et al., 2018). However, high concentrations of acetate decreased inflammation and barrier permeability in organoid-derived cultures from patients with ulcerative colitis (Deleu et al., 2023). Our data shows that butyrate can prevent both transcellular and paracellular LPS-induced permeability in *in vitro* models of the gut and blood-brain barrier, while also enhancing baseline gut barrier integrity in T84 cells. These findings agree with those of Li et al. showing that that butyrate can restore BBB and ZO-1 levels in traumatic brain injured mice (Li et al., 2016) and with Beisner and colleagues who observed that sodium butyrate attenuated western-diet-induced gut barrier dysfunction (Beisner et al., 2021). Regarding propionate, it has been found to improve intestinal epithelial barrier dysfunction in Parkinson's disease patients (Huang et al., 2021). In this current study, we also show that propionate improves brain transcellular and gut paracellular permeability but does not affect transcellular gut integrity. We previously demonstrated that propionate influences the abundance of tight junction proteins, such as ZO-1 and claudin-5, in brain endothelial cells (Knox et al., 2022). ZO-1 is recognized for its role in modulating paracellular permeability, while claudin-5, which is primarily associated with brain endothelial, rather than gut epithelial cells, plays a dual role in regulating both paracellular and transcellular permeability by mediating ion passage across tight junctions (Greene et al., 2019). Therefore, the modulation of claudin-5 that is specifically observed in bEnd3 cells may account for the differences in the effects of propionate on gut and brain barriers.

An important question arising from these observations concerns the underlying mechanisms responsible for the alterations in SCFA levels. We thus used a comprehensive bioinformatic analysis of colonic tissue and caecal content metabolomic datasets to further understand why SCFAs are reduced by acute stress. This was essential to explore how stress can affect the gut and modify the availability of microbial metabolites which in turn can modulate host physiology. The reduction in simple carbohydrates and SCFAs, along with an increase in pyruvate and succinate in the gut lumen, could indicate a disruption in microbial fermentation processes, alterations in substrate availability, or increased SCFA absorption. The increase of ribitol suggests that under acute stress conditions, some gut microbiota may shift their fermentation pathways, producing polyols instead of SCFAs when the gut environment becomes less favorable for SCFA production, such as through changes in pH, or other stress-induced metabolic shifts. In this context, some microbially-related enzymes involved in Krebs or methylcitrate cycles were also altered by acute stress in a microbiome-dependent manner. The difference between conventional, germ-free and colonized germ-free mice also indicates the importance of microbial content of the gastrointestinal tract in influencing the stress response, which is also shown in the literature (Neufeld et al., 2011; Sudo et al., 2004). For instance, here we show that oxalate, which is usually increased following stress, is reduced in the absence of a gut microbiota, whereas 2-methylcitrate cycle, which is necessary to metabolize and detoxify propionate, is reduced only in conventional mice, which could lead to concomitant accumulation of propionyl-CoA, a toxic metabolite that inhibits cell growth (Kumar et al., 2023; Limenitakis et al., 2013; Weljie et al., 2015). Since SCFAs are a key energy source for colonocytes, the absence of significant changes in metabolic compounds within these cells, coupled with an increase in microbial metabolism, suggests plausible explanations for acute stress-induced alterations in SCFAs. These include either an enhancement of microbial metabolism, or an increased diffusion of SCFAs across the gut epithelium, combined with an impaired ability of colonocytes to effectively utilize SCFAs (den Besten et al., 2013; Litvak et al., 2018). Future analysis of circulating SCFAs should be considered to confirm this hypothesis.

There are also some important limitations to consider. Although *in vitro* studies are essential to understand the effects of stress-driven changes in microbial metabolites, such as SCFAs, T84 cells are a valuable tool for high-throughput screening and experimental manipulation. These cells allow for more controlled analysis of the mechanisms involved, which may be more difficult to capture in primary colonocytes due to their more variable and differentiated characteristics. However, to fully understand those effects, future research involving first primary colonocytes and *in vivo* models analysing systemic SCFAs levels with both male and female mice will be needed to explore its effects on the whole organism and the microbiota-gut-brain axis, while also considering potential possible sex differences. Our findings provide a better understanding of the relationship between acute stress and the microbiota-gut-brain axis, with SCFAs implicated as effectors of intestinal permeability alterations. Further work is warranted to understand what happens when these acute stress exposures are experienced repeatedly and chronically as the associated adaptive or maladaptive consequences emerge.

Modulating the gut microbiota is essential to consider for the future development, prevention and maintenance of neuropsychiatric disorders and to counteract impairment of t intestinal function (E. Schneider et al., 2024; Seethaler et al., 2022). Acute stress response has been found to modulate the gut microbiota (Ma et al., 2023; Tofani et al., 2024) Moreover, other research has also demonstrated that SCFAs can alleviate stress symptoms both in mice and humans (Dalile et al., 2020; van de Wouw et al., 2018). However, to date, most studies focus on chronic stress rather than a single acute stressor. Here, we demonstrate that SCFAs are acute stress-related microbial metabolites, which can prevent LPS-induced brain and gut barrier permeability. These results provide a mechanistic understanding of microbial modulation of gut and brain barriers and pave the way for interventions to counteract the detrimental impact of stress.

## CRediT authorship contribution statement

**Cristina Rosell-Cardona:** Writing – review & editing, Writing – original draft, Visualization, Methodology, Investigation, Formal analysis, Data curation, Conceptualization. **Sarah-Jane Leigh:** Writing – review & editing, Writing – original draft, Visualization, Methodology, Investigation, Formal analysis, Data curation, Conceptualization. **Emily Knox:** Writing – review & editing, Writing – original draft, Visualization, Methodology, Investigation, Formal analysis, Data curation, Conceptualization. **Emanuela Tirelli:** Writing – review & editing, Investigation. **Joshua M. Lyte:** Writing – review & editing, Methodology, Conceptualization. **Michael S. Goodson:** Writing – review & editing, Funding acquisition. **Nancy Kelley-Loughnane:** Writing – review & editing, Funding acquisition. **Maria R. Aburto:** Writing – review & editing, Supervision, Funding acquisition. **John F. Cryan:** Writing – review & editing, Supervision, Funding acquisition, Conceptualization. **Gerard Clarke:** Writing – review & editing, Supervision, Funding acquisition, Conceptualization.

## Resource availability

### Lead contact

Further information or requests for resources and reagents should be directed to the lead author, Gerard Clarke (g.clarke@ucc.ie).

### Materials availability

This study did not generate any unique reagents or materials.

### Data and code availability

• Untargeted metabolomics presented in this paper comprise post-hoc analysis of a dataset previously published (caecal contents, https://doi.org/10.21228/M89M7J; Project ID: PR001945; colonic mucosa, https://doi.org/10.21228/M89M7J; Project ID: PR001945) 7.
• In vitro data generated in this paper will be shared by the corresponding authors upon request. Any additional information required to reanalyze the data reported in this paper is available from the lead contact upon request.

## Declaration of competing interest

The authors declare the following financial interests/personal relationships which may be considered as potential competing interests: The research was conducted in the APC Microbiome Ireland which is funded by 10.13039/501100001602Science Foundation Ireland (now Research Ireland, SFI/12/RC/2273_P2).

This project reports financial support was provided by 10.13039/100015464European Office of Aerospace Research and Development, Air Force Office of Scientific Research and 711 Human Performance Wing, 10.13039/100006602Air Force Research Laboratory.

The views expressed are those of the authors and do not reflect the official views of the United States Air Force, nor the Department of Defense. Mention of trade names, commercial products, or organizations do not imply endorsement by the U.S. Government (Distribution Statement A. Approved for public release. Distribution is unlimited. Case Number: AFRL-2024-6130, Nov 20, 2024).

Sarah-Jane Leigh reports financial support was provided by 10.13039/501100002081Irish Research Council Postdoctoral Fellowship. Maria R. Aburto reports financial support was provided by 10.13039/501100000781European Research Council. Maria R. Aburto reports financial support was provided by 10.13039/501100001602Science Foundation Ireland Public Fellowship Programme.

John F. Cryan reports a relationship with Bromotech that includes: speaking and lecture fees. John F. Cryan reports a relationship with Reckitt that includes: research funding grants. John F. Cryan reports a relationship with Nutricia Research BV that includes: research funding grants. John F. Cryan reports a relationship with DuPont Nutrition & Biosciences that includes: research funding grants. John F. Cryan reports a relationship with Nestle that includes: speaking and lecture fees; research funding grants. Gerard Clarke reports a relationship with 10.13039/100008897Janssen Pharmaceuticals Inc that includes: speaking and lecture fees. Gerard Clarke reports a relationship with Probi AB that includes: speaking and lecture fees. Gerard Clarke reports a relationship with Boehringer Ingelheim GmbH that includes: speaking and lecture fees. Gerard Clarke reports a relationship with Apsen Farmacêutica SA that includes: speaking and lecture fees. Gerard Clarke reports a relationship with 10.13039/100007082Pharmavite 10.13039/100023970LLC that includes: research funding grants. Gerard Clarke reports a relationship with 10.13039/501100003144Fonterra Research and Development Centre that includes: research funding grants. Gerard Clarke reports a relationship with Tate and Lyle that includes: research funding grants. Gerard Clarke reports a relationship with Nestle that includes: research funding grants. Gerard Clarke reports a relationship with Yakult Central Research Institute for Microbiological Research that includes: consultancy. Gerard Clarke reports a relationship with Heel Pharmaceuticals that includes: consultancy, speaking and lecture fees. Gerard Clarke reports a relationship with Zentiva Group that includes: consultancy.

This support neither influenced nor constrained the contents of this manuscript.

All other authors declare that they have no known competing financial interests or personal relationships that could have appeared to influence the work reported in this paper.

## Data Availability

Data will be made available on request.

## Appendix A Supplementary data

Supplementary Figure 1: Other short-chain fatty acid concentrations in the gut lumen following acute restraint stress. Caecal content concentrations of (A) 2-methylbutyric acid, (B) isobutyric acid, (C) valeric acid, (D) isovaleric acid and (E) hexanoic acid. Data as mean ± SEM, overlaid individual values (*n* = 7 mice/group); analyzed by two-way general linear modeling followed by Tukey-adjusted post hoc comparisons. ∗p < 0.05, ∗∗p < 0.01, and ∗∗∗p < 0.001.

Supplementary Figure 2: Other short-chain fatty acid substrate concentrations in the gut lumen following acute restraint stress. (A) Dietary fiber is metabolized by the gut bacteria into various metabolites such as butyrate, acetate, and propionate. Major pathways include Wood-Liungdahi, acylate, succinate, and propanediol pathways. Metabolites tested in this study are highlighted in purple. Caecal content concentrations of (B) oligosaccharides, (C) disaccharides, (D) other related metabolites and (E) other microbial metabolites produced from monosaccharides. Data as mean ± SEM, overlaid individual values (*n* = 7 mice/group); analyzed by two-way general linear modeling followed by Tukey-adjusted post hoc comparisons. ∗p < 0.05, ∗∗p < 0.01, and ∗∗∗p < 0.001.

Supplementary Figure 3: Energy-related metabolite concentrations in the colonic mucosa following acute restraint stress. (A) Energy metabolism in intestinal epithelial cells is able to utilize short-chain fatty acids as well as other microbially-produced metabolites as substrates. Major pathways include the methylcitrate cycle and TCA cycle glyoxylate shunt. Colonic mucosal concentrations of (B) TCA cycle substrates, (C) microbially-associated TCA cycle substrates and (D) TCA cycle components. Data as mean ± SEM, overlaid individual values (*n* = 7 mice/group); analyzed by two-way general linear modeling followed by Tukey-adjusted post hoc comparisons. ∗p < 0.05, ∗∗p < 0.01, and ∗∗∗p < 0.001.

Supplementary Figure 4Short-chain fatty acids did not alter actin directionality (A) Representative 60x confocal images of phalloidin-stained actin following butyrate, propionate, or acetate treatment with and without LPS, coherency coefficients of the filamentous actin in the 60x confocal image in T84 cells following 48hr (B) butyrate, (C) propionate, and (D) acetate treatment with and without LPS for the last 24hrs of incubation. Data are mean ± SEM two-way ANOVA followed by Tukey's post hoc. Scale bar: 20 μm.

Supplementary Figure 5: Cell viability after short-chain fatty acids treatment (A) MTT (3-[4,5-dimethylthiazol-2-yl]-2,5 diphenyl tetrazolium bromide) assay of T84 cells and bend.3 cells following butyrate (A,D), propionate (B, E), or acetate (C,F) treatment. Data are mean ± SEM one-way ANOVA followed by Tukey's post hoc. ∗p < 0.05, ∗∗p < 0.01, ∗∗∗p < 0.001 metabolite compared to 0 μM control.

Supplementary Figure 6: Lipopolysaccharides optimization (A) FITC dextran 4 kDa permeability assay in T84 cells with different concentrations of LPS, (B) percentage of T84 cells alive with LPS and positive controls (DMSO), (C) percentage of T84 cells dead with LPS and positive controls (DMSO). Data are mean ± SEM one-way ANOVA followed by Tukey's post hoc. ∗p < 0.05, ∗∗p < 0.01, ∗∗∗p < 0.001 compared to untreated.
