# Supplementary material for: Acute stress-induced alterations in short-chain fatty acids: Implications for the intestinal and blood brain barriers
Source: Brain Behav Immun Health. 2025 Apr 17;46:100992. doi: 10.1016/j.bbih.2025.100992 (PMC12159890; doi:10.1016/j.bbih.2025.100992)

SUPPLEMENTARY FIGURE 1
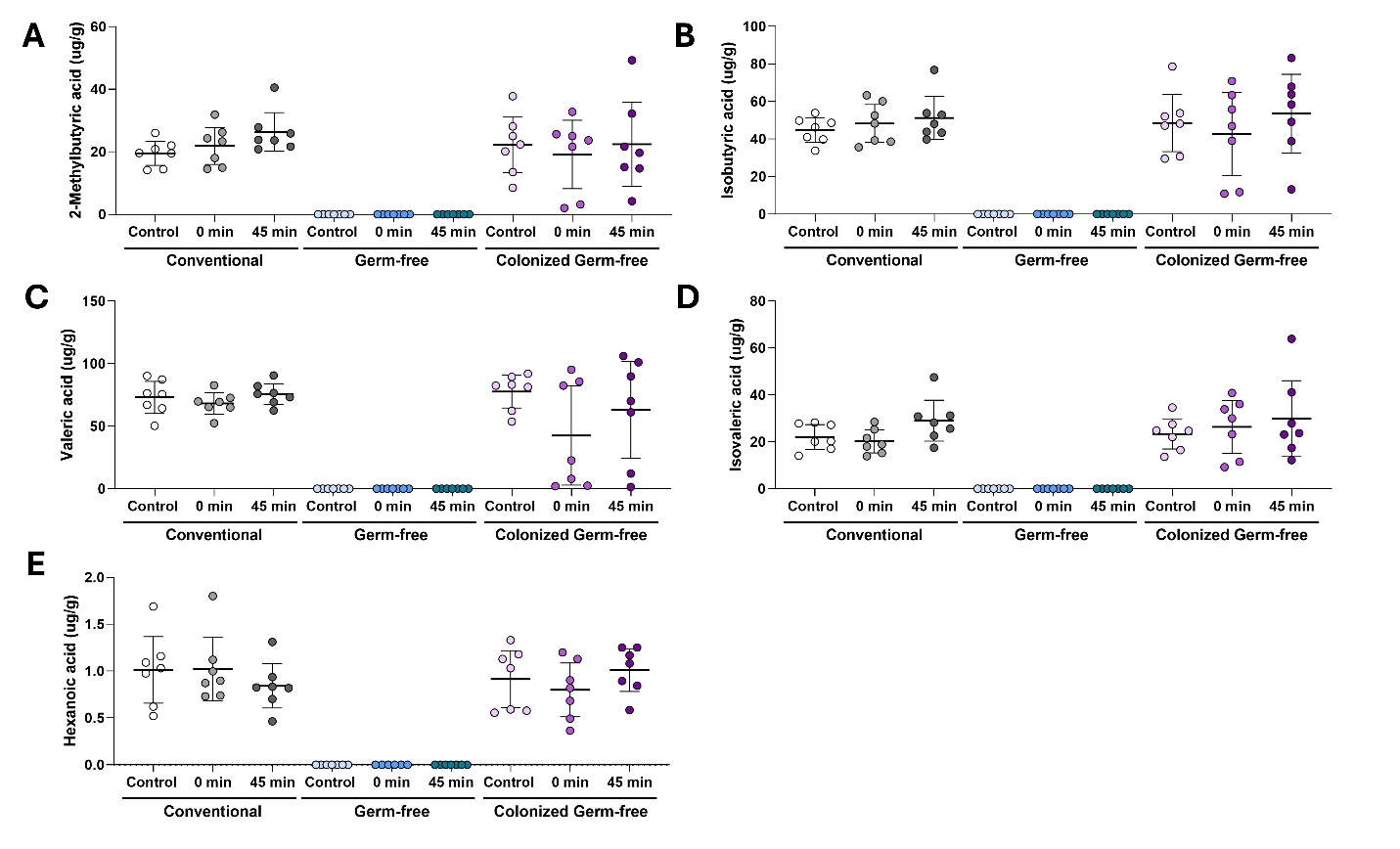


# SUPPLEMENTARY FIGURE 2
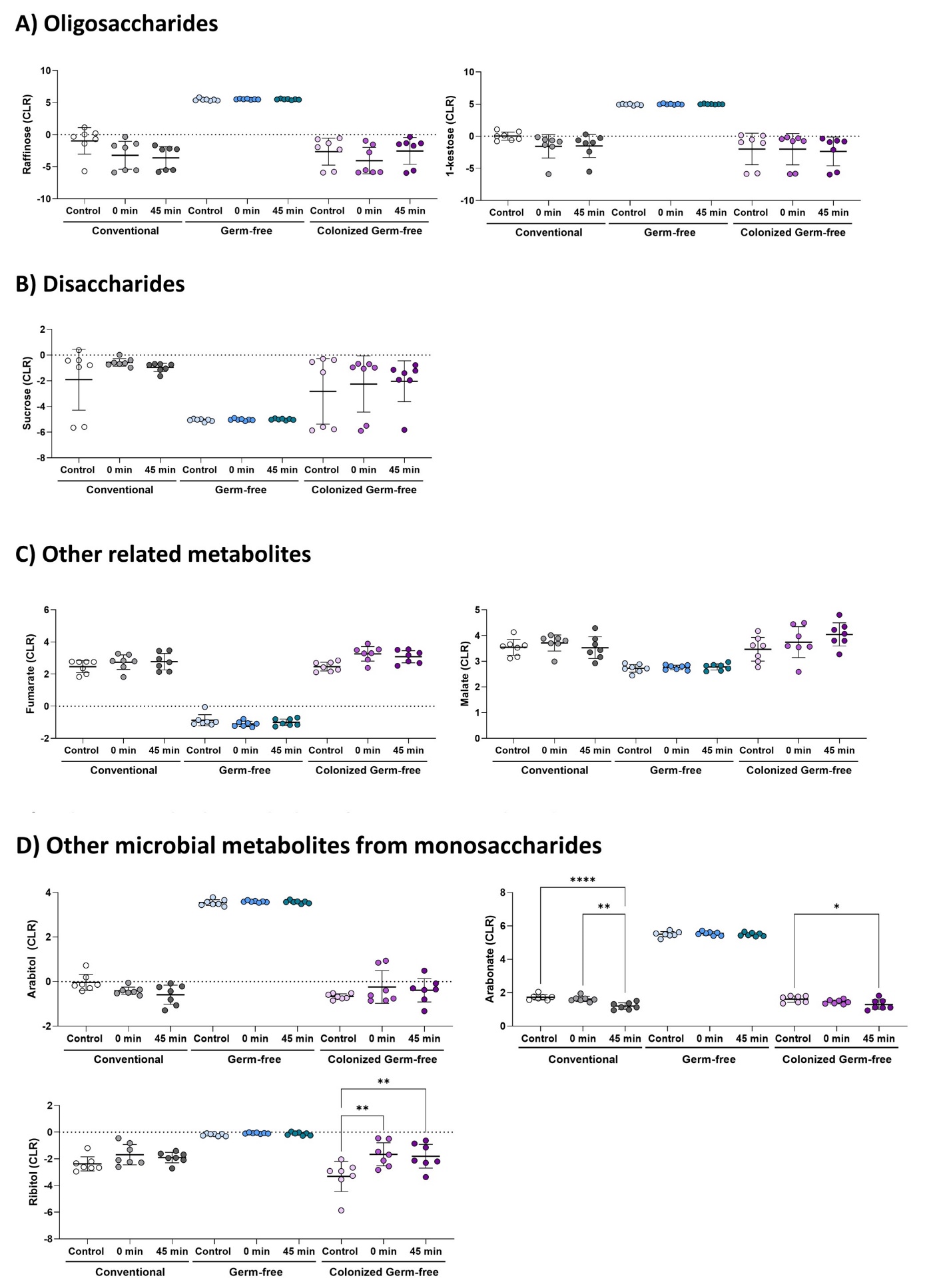


# SUPPLEMENTARY FIGURE 3
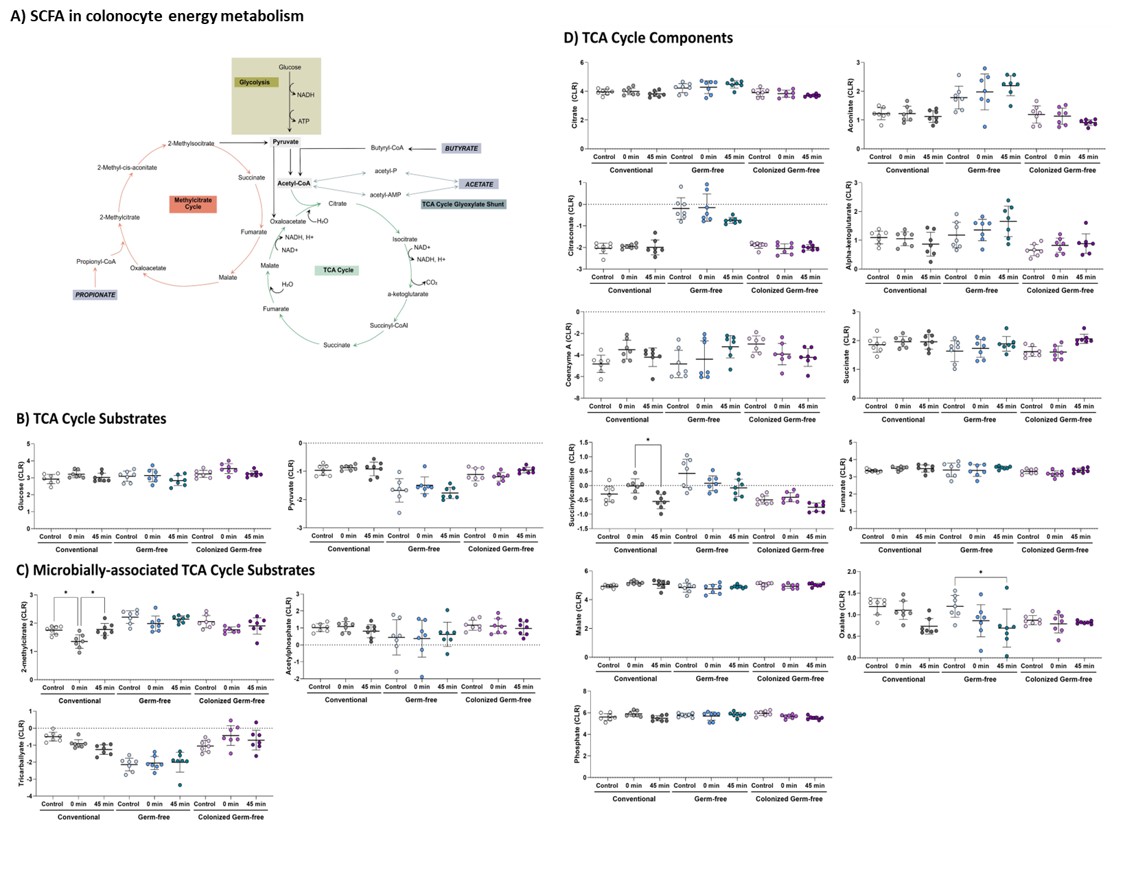
SUPPLEMENTARY FIGURE 4
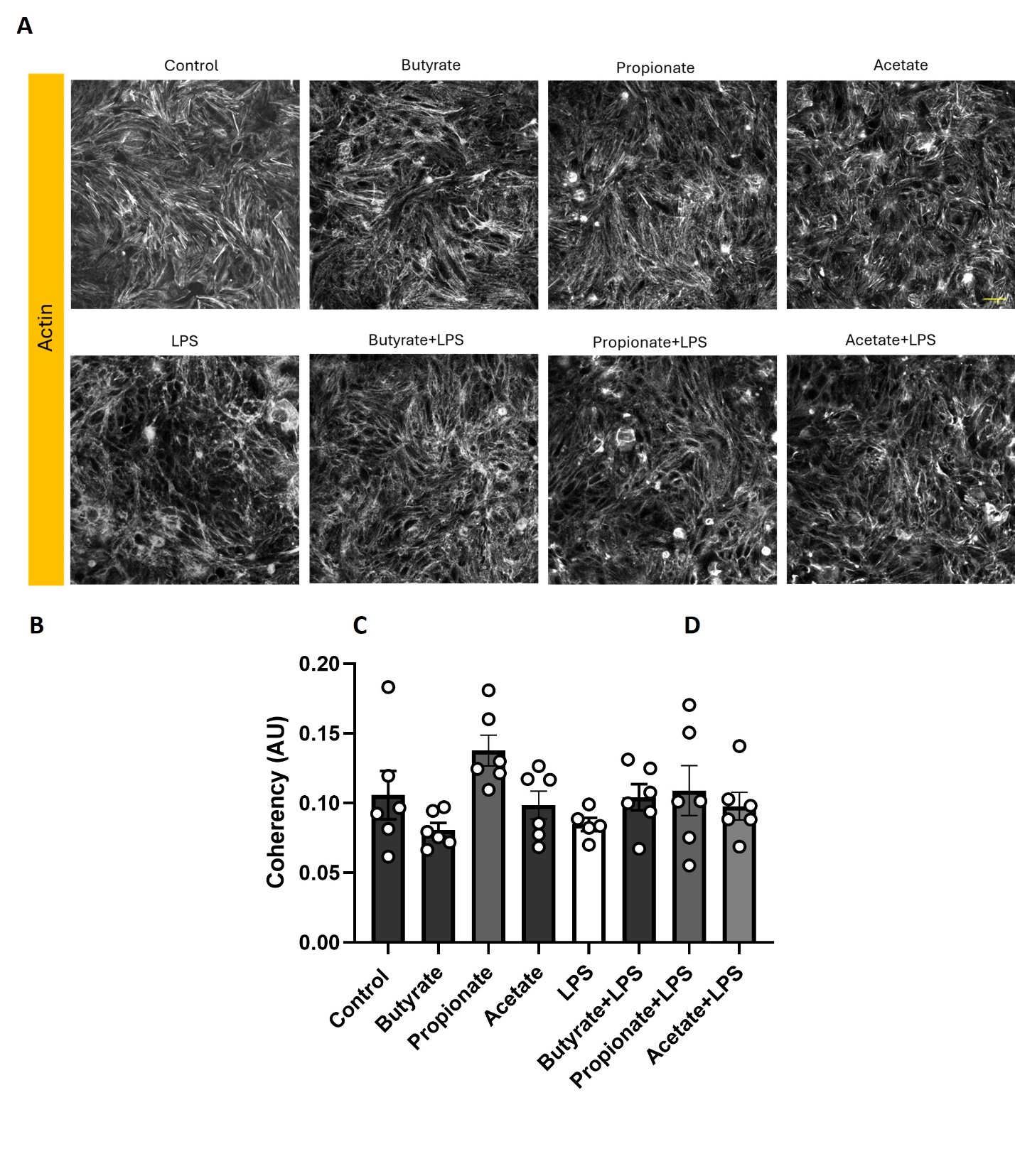


# SUPPLEMENTARY FIGURE 5
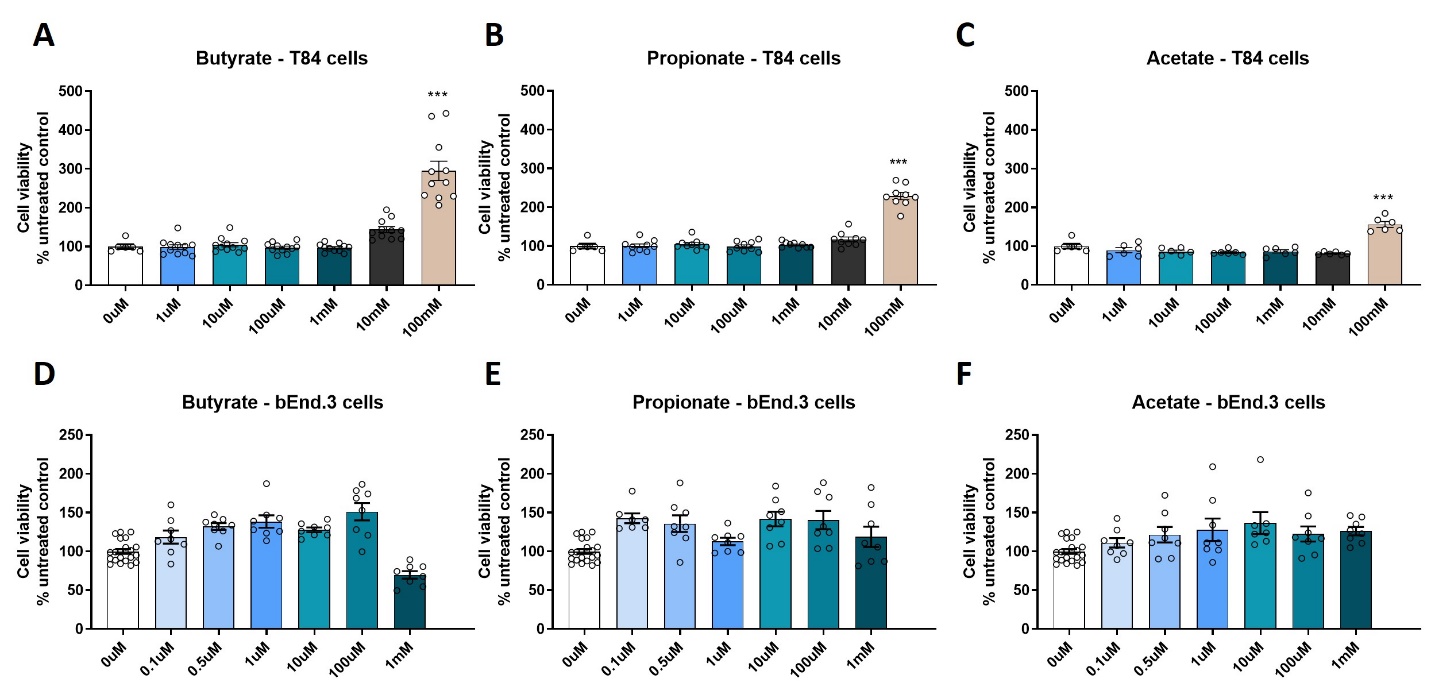


# SUPPLEMENTARY FIGURE 6
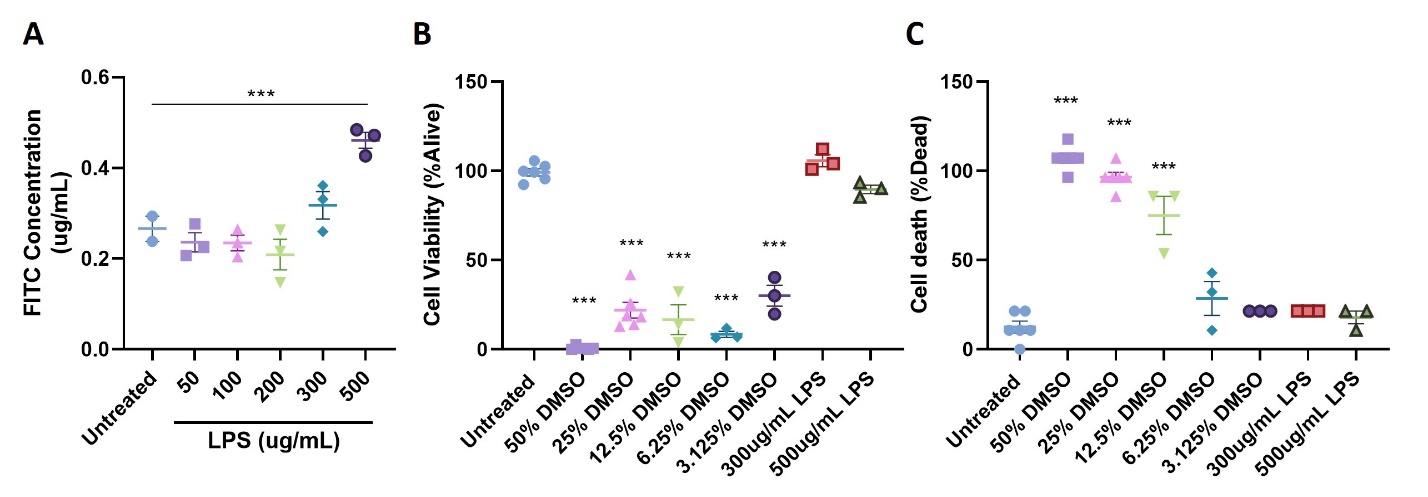

Supplement: Multimedia component 1 [file mmc1.docx]
